# Community as a source of health in three racial/ethnic communities in Oregon: a qualitative study

**DOI:** 10.1186/s12889-015-1462-6

**Published:** 2015-02-12

**Authors:** Carolyn A Mendez-Luck, Jeffrey W Bethel, R Turner Goins, Marc B Schure, Elizabeth McDermott

**Affiliations:** College of Public Health and Human Sciences, Oregon State University, Corvallis, OR 97331 USA; Department of Social Work, Western Carolina University, Cullowhee, NC 28723 USA; Veterans Affairs—Health Services Research & Development, Seattle-Denver Center of Innovation for Veteran-Centered and Value-Driven Care, Seattle, WA 98108 USA

**Keywords:** American Indian, Latino, Migrant and seasonal farmworkers, Community health, Trauma

## Abstract

**Background:**

A 2011 report by the Oregon Health Authority and the Department of Human Services documented disparities in its Latino and American Indian populations on multiple individual-level health indicators. However, research is lacking on the social contexts in which Latinos and American Indians in Oregon live and how these environments influence the health of communities as a whole. To help fill this gap, this study sought to contextualize the social environments that influence the health of Latinos and American Indian residents in three Oregon communities.

**Methods:**

Guided by an ecological framework, we conducted one-time semi-structured qualitative interviews with 26 study participants to identify the prominent health-related issues in the communities and to examine the factors that study participants perceived as enabling or inhibiting healthy lifestyles of community residents. We used a grounded theory approach to perform content and thematic analyses of the data.

**Results:**

Study participants identified preventable chronic conditions, such as diabetes, obesity, and hypertension, as the most pressing health concerns in their communities. Results showed that traditional and cultural activities and strong family and community cohesion were viewed as facilitators of good community health. Poverty, safety concerns, insufficient community resources, and discrimination were perceived as barriers to community health. Three themes emerged from the thematic analyses: social connectedness is integral to health; trauma has an ongoing negative impact on health; and invisibility of residents in the community underlies poor health.

**Conclusions:**

This study’s findings provide insight to the social contexts which operate in the lives of some Latinos and American Indians in Oregon. While participants identified community-level factors as important to health, they focused more on the social connections of individuals to each other and the relationships that residents have with their communities at-large. Our findings may also help to explain how the intra- and inter-personal levels, the community/institutional level, and the macro level/public policy contexts can serve to influence health in these communities. For example, trauma and invisibility are not routinely examined in community health assessment and improvement planning activities; nonetheless, these factors appear to be at play affecting the health of residents.

## Background

Latino and American Indian adults suffer from excess morbidity as a result of multiple factors such as lower socioeconomic status and lower levels of physical activity. U.S. Census data indicate that 23% of persons of Hispanic origin live below the poverty level while 27% of American Indians lived below the poverty level, the highest national poverty rate for all races and ethnicities [1]. The Institute of Medicine has identified these two populations as disparities populations with respect to health care access [2]. Research has consistently indicated that Latinos and American Indians compared to their white counterparts engage in significantly less physical activity [3,4]. Although these individual factors are critical determinants of health, researchers have increasingly emphasized how social environments affect individual health and health disparities. Similar people tend to live within geographical proximity of each other, whether purposefully to share a common culture or because of a lack of resources. Community resources have been shown to be important factors that influence community residents’ ability to be healthy. Communities that are deficient in these structural areas are more likely to have higher prevalence of preventable disease and higher proportions of ethnic and racial minorities compared to communities with greater resources [5,6].

The Oregon Health Authority and the Department of Human Services collaborated on a 2011 report of the state of equity in health among all Oregonians. The report documented disparities in its Latino and American Indian populations on multiple individual-level health indicators, such as use of preventive services [7]. However, research is lacking on the social contexts in which Latinos and American Indians in Oregon live and how these environments influence the health of communities as a whole. To help fill this gap, we conducted a qualitative study in three communities to identify the prominent health-related concerns within a socio-cultural context and to examine the factors that community leaders perceived as enabling or inhibiting healthy lifestyles of community residents. Our main research questions were, “What health problems do community members perceive as the biggest threats to the community’s health; and, which characteristics of the communities do members perceive as supporting or impeding healthy living?”

## Methods

We deemed qualitative research methods as the best approach in addressing the aims of this study. Through semi-structured interviews and an ecological framework, we sought to explore the lived experiences of Latino and American Indian community leaders and residents actively engaged in their communities. An ecological framework offers a broad perspective of health promotion and integrates multiple levels of factors that influence health—intra- and interpersonal, community/institutional, and macro level/public policy [8].

### Study sites

We conducted our study in three communities in Oregon that had documented high rates of poverty and health disparities and high concentrations of ethnic minority residents [9]. The first community was an urban neighborhood located in the Portland, Oregon metropolitan area. The neighborhood is comprised of approximately 5,000 households, of which 20% have incomes below 100% of the Federal Poverty Level and 20% are of Latin American origin [10]. The second community was a rural agricultural area in the Willamette Valley. The Willamette Valley is comprised of parts of 10 counties in western Oregon, where 23,000 migrant and seasonal farmworkers (MSFW) reside in one county alone [11]. Similar to national estimates, the majority of the MSFWs in the Willamette Valley and Oregon come from Mexico and have incomes below the poverty level [11]. The American Indian community was located on a reservation in a rural county where 16.5% of persons have incomes below the federal poverty level [12]. More detailed information is not presented to protect the identity of the community.

### Sample recruitment and characteristics

Community leaders from the three communities were recruited to participate in this study. We defined community leaders as community residents and/or employees of community-based organizations that served the geographic area and/or Latino/American Indian communities. Study participants were initially identified through the researchers’ existing relationships and knowledge of local community-based organizations, word of mouth, and discussions with the Oregon State University Extension Service. Subsequent study participants were recruited using snowball sampling, which used referrals from other participants [13]. Adults who met the following criteria at the time of interview were eligible to participate in the study: 1) at least 18 years old, 2) lived or worked in the community, and 3) considered themselves knowledgeable about the community.

We aimed to interview 10 study participants in each community for a total of 30 interviews which we believed would be adequate to reach theoretical saturation for the focused topics in this study. However, we ended enrollment early after determining that no new findings were emerging from the analysis of our interviews. Thus, a total of 26 participants participated in this study (Table 1). The majority of participants were female (n = 20), Latino (n = 16), and were employees of community-based organizations (n = 23) such as local law centers, health clinics, and cultural centers. On average, participants had spent 14 years engaged with their respective communities either working or living in these communities.

**Table 1:** **Description of sample**

	**Rural Latino Community**	**Urban Latino Community**	**American Indian Community**	**Total**
	**N = 7**	**N = 10**	**N = 9**	**N = 26**
	**Mean (SD)**	**Mean (SD)**	**Mean (SD)**	**Mean (SD)**
Age (years)	48 (12)	49 (9)	51 (8)	49 (9)
Time Involved with Community (years)	10 (9)	12 (6)	21 (22)	14 (13)
	*n*	*n*	*n*	*n*
Female	4	10	6	20
Immigrant	5	8	n/a	13
American Indian	0	0	9	9
Latino	7	9	0	16
White, non-Latino	0	1	0	1

### Data collection

We obtained informed consent from the study participants using procedures approved by the institutional review boards of Oregon State University and the Northwest Portland Area Indian Health Board.

Data were collected through semi-structured interviews using an interview guide that covered three topics: meaning of good health, enabling and inhibiting factors of good health, and most important health-related concerns facing the community. Probing questions were also used to elicit a richer set of responses for each topic. We specifically developed a set of questions that addressed healthy living on each level of influence from the ecological framework (Table 2).

**Table 2:** **Interview guide questions and level of Ecological Framework targeted**

**Question**	**Level of ecological framework targeted**
Introduction	
1. How long have you worked/lived in [name of agency or community]?	N/A
Meaning of Good Health	
First, I want you to think about your own health and life.	Intra-Personal
2. What do you consider “good health”?	
3. What do you think it means to live a healthy lifestyle?	
Now I want you think about [insert name of community]. I’d like for you to think about the community residents that you see every day and interact with either personally or for your job. I also want you to think about the community’s physical environment, stores, restaurants, health and human services, community programs, and so on.	Community/Institutional
4. What does it mean for a community to be “healthy”?	
5. Do you consider your community to be healthy?	
6. How would you rate the health of this community as a whole on a scale of 1 to 5, with 1 being poor and 5 being excellent?	
*Probe:* What factors did you take into account with your rating?	
Enabling and Inhibiting Factors of Good Health	
7. What are the values around being healthy in this community?	Inter-Personal
8. What are the unique characteristics (strengths) in [insert name of community] that help residents in being healthy?	
*Probe:* What other characteristics within this community support being healthy?	
9. What are the unique characteristics (weaknesses) in [insert name of community] that prevent residents from being healthy?	
*Probe:* What other characteristics within your community discourage residents from being healthy?	
10. Which of these characteristics, good and not good, are specific to the [insert Native/MSFW/Latino] culture in this community?	
11. What are the biggest (or most important/pressing) health-related concerns in this community?	Community/Institutional
*Probe:* In your point of view, what are the factors that contribute to these health problems?	
12. What resources are available or in place in [insert name of community] to support healthy living among the residents?	Macro Level/Public Policy
*Probe:* How much are these resources used by community residents?	
*Probe:* How widely known are these resources by community residents?	
Wrap Up	
13. What are other important health issues in this community that we have not discussed so far?	All

The interviews were conducted by the faculty researchers or graduate research assistants trained in field research and qualitative interviewing techniques. The majority of interviews took place in person (n = 15), and the rest were conducted by telephone. All interviews were tape-recorded, conducted in English or Spanish, and lasted an average of 45 minutes. The audio tapes were transcribed verbatim by a professional transcription service and cross-checked for accuracy by the investigators.

### Data analyses

We entered the interview transcripts into Atlas.ti (Version 7.1), a qualitative data management software program [14]. We analyzed the data in the language of the interview using a grounded theory approach, which involved an iterative process of content analyses and taxonomic organization [15]. The purpose of the content analyses was to become familiar with the breadth and scope of the data. To do this, we coded the responses to each question and then grouped the codes into broader categories around the three topic areas in the interview guide. The taxonomic organization involved breaking down the transcripts into fragments of text, clustering text around single words or phrases, coding the clusters of text, organizing those clusters by concepts and then identifying thematic content from these concepts. Three graduate student researchers coded the data and the three faculty researchers reviewed all of the coding. The entire research team reconciled the coding to reach consensus on the coding after each pass through the data. When differences in coding occurred, the coders challenged each other’s interpretation of the code and the rationale behind the coding decision. A disputed coding decision was resolved by returning to the original transcript to find additional evidence for substantiating the coded text. If no additional evidence was found or if the coding could not be substantiated, the specific text was unlinked from the code. If a text was coded differently by each coder yet both codes were substantiated, the coded text was left alone. Upon completion of the taxonomic organization, the faculty researchers identified thematic content.

Spanish-language quotes were subsequently translated into English for use in this article. The original quotes in Spanish are available from the primary author upon request. The quotes used in this article are verbatim. We have used pseudonyms to protect the identity of study participants.

## Results

### Content findings

#### Meaning of good health

Most study participants believed that good health on an individual level meant being “physically active” and/or engaging in “exercise” and “eating well”. According to participants, eating well included a diet that was rich in fruits and vegetables and limited in fatty or fried foods. Some participants believed that good health meant “having good emotional health”, “mental wellbeing”, or being a “well-rounded person emotionally, spiritually and physically”. Participants also believed that good health meant having a “balanced life”, “not being overly stressed”, “feeling good about oneself”, and having “strong” family and personal relationships.

Study participants believed that good health on a community level meant having resources to offer its residents. They viewed goods and services as a marker of good community health, such as the availability and accessibility of health and social services, recreational activities, and affordable healthy foods. Participants also believed that a community had good health if it was safe for residents to live, walk, or play. They discussed safety in terms of crimes against persons and the existence of gang and drug activity, but also in terms of the physical environment, such as the lack of sidewalks and adequate lighting.

#### Enabling and inhibiting factors of good health

The majority of study participants reported that they did not consider their respective communities to be healthy. The most common reasons cited were high rates of poverty in the community, lack of community resources that supported health-promoting activities, discrimination, and high rates of chronic conditions, such as type 2 diabetes and obesity. Study participants listed these and other factors related to socio-economic status, such as lack of education, financial difficulties, and unemployment, as unique characteristics of their communities that inhibited residents from being healthy. They also identified safety concerns, such as gangs, domestic violence, and drug and alcohol abuse as community problems that prevented or limited residents’ ability to lead healthy lifestyles. One study participant, Laura, shared her views on inhibiting factors that echoed the sentiments of other participants. At the time of the interview, Laura was 57 years old and had been working for seven years on educational programming for the Latino adults in her community. She stated:

I am realizing that this area is a very dangerous zone. Gangs, right? That can also influence… in some ways well the families don’t leave their homes. The families that want to take care of their health and it’s night and around here they can [be] assault[ed]…It’s a factor in this area that really harms the mental health, above all, of our children.

Additionally, participants of the Latino communities perceived that residents faced language and cultural barriers as well as lack of legal status which kept residents from being healthy.

Despite the sentiments of Laura and others, all study participants identified at least one specific resource available in their communities that they believed enabled a healthy lifestyle. These resources included health clinics, food pantries, family resource centers, and health, nutrition or exercise classes. The majority of participants viewed the factors which promoted community health within the context of family and community cohesion. Participants perceived that the community’s health-related values were centered on providing for the family and on living a long life with family. These views were illustrated in the interview with Richard, the director of a non-profit organization in the community. He stated:

I think from what I have seen also, it’s around family. The idea of being healthy is connected to the value of having a strong family, having a happy family, being able to provide for the basic necessities [if] they can provide what their children need, then…their children are being healthy and their family is being healthy.

Many participants also viewed traditional and cultural activities as positive, health-promoting practices, although the specific activities differed by community. Several participants of the Latino communities indicated that preparing traditional meals was healthy because the meals reinforced residents’ culture and unity, which were thought to be important components of health for both the individual and the community as a whole. Several participants of the American Indian community shared similar beliefs in that cooking and socializing with others promoted good health and strengthened social ties. However, many participants of the American Indian community believed that other cultural activities, such as fishing, and gathering roots and berries, promoted physical activity and community unity. No participant of the Latino communities mentioned these same activities as health-promoting cultural practices. As one participant from the American Indian community commented, “that’s what I see as healthy… exercising some of the traditions which we do. We go to the celebrations. We do the healthy things [together] that enrich our soul”. Nonetheless, most study participants, regardless of the community they represented, saw cultural practices as vehicles for bringing families and friends together, strengthening those social ties, and creating a more unified community as a result.

#### Most important health-related concerns

Study participants identified preventable chronic conditions as the most important or pressing health concerns in their communities. Type 2 diabetes was most commonly mentioned by participants, followed by obesity, hypertension, and high cholesterol. Other pressing health-related issues identified included depression, suicide, hunger, and food insecurity. Participants reported an unbalanced or poor diet and lack of exercise as the major factors attributing to these health problems in the community. Participants also cited stress and trauma as contributors to poor health in the community.

### Thematic findings

Three themes emerged from the grounded analysis of healthy living discussions: Social connectedness is integral to health, trauma has an ongoing negative impact on health, and invisibility of residents in the community underlies poor health.

#### Social connectedness

The first theme that emerged from our thematic analysis was the participants’ views that being socially integrated with family, friends, and the community at-large supported an individual’s attempts to lead a healthy life. We found that almost all study participants believed that having dense social networks were key motivators for pursuing a healthy lifestyle and were foundational for good health. One key informant from the rural Latino community remarked, “That you’re able to have [people] surround you that love you and you are able to love, you know, people who support you when you’re really sick or sad. For me, that is health”. The importance of being socially connected and its connection to health was a notion that was shared by many study participants from all communities.

We found that social connectedness was an important feature of the family but also of the larger community. Some participants believed that social connections helped pass the “healthy word” along to others and encouraged participation in health-promoting activities. However, being socially connected also meant “keeping an eye out” for each other, “checking in” with others, and helping people in need in the community at-large. The following excerpt represented the sentiments shared by many study participants about being socially connected beyond the immediate family:

I think it’s important for them [residents] to stay healthy, not just in their family but in their community….Everybody’s concerned about sharing the healthy information to people so it is something that….that they understand needs to be thought about in order to have a healthy community, a healthy family, healthy children.

This excerpt demonstrated participants’ views that social connections are fundamentally important to good health at the family and community level. Conversely, lack of social connections or “bad blood” with others can be harmful to health, according to study participants. As one participant stated, “sometimes there are people who close themselves off when they have a problem or they don’t have a solution or don’t know where to get solutions or who to talk to and that also may affect [them] emotionally or mentally”.

#### Trauma

The second theme that emerged from our grounded analysis of healthy living discussions was that personal and community-wide traumatic experiences negatively impacted residents’ health and sense of wellbeing. While the type of trauma described differed slightly across communities, study participants from all three communities shared the view that both present-day and past traumatic experiences affected the current health of their residents.

In the American Indian community, several study participants indicated that they lowered their health ratings of their community because of the trauma experienced within the community. Study participants discussed trauma in terms of community-level historical events and in terms of one’s own family where the behavior of one member affected another or the family as a whole. Examples of community-wide trauma that were considered as major contributing factors to the American Indian community’s health problems included having had tribal lands taken away, forced residential schooling, and the betrayal of the U.S. government. Examples of family-level trauma included alcoholism, drug abuse, and personal violence.

Study participants’ discussions indicated that trauma at the community and family levels was actually intertwined and viewed as intergenerational. Veronica, an American Indian study participant worked at a community health center and was asked what she meant by “[inter]generational trauma”. She responded,

Well, the loss of their language, the loss of their way of life, the violence perpetrated on them, you know. And I mean we’re talking hundreds of years, but the violence perpetrated to get them into reservations, taking away their self-respect. Violence was committed on them by others, perpetuated on each other by themselves, alcoholism, you know, all those kinds of things that break families and has never been addressed or talked about.

Other American Indian community study participants echoed these sentiments, suggesting that trauma was a form of violence that destroyed traditional customs and foods, the environment, and the structure of the family. The cumulative impact of these losses was seen as having a detrimental effect on the health of community residents, leading to “depression”, “poor eating habits”, “violence”, and “poverty”, among other problems.

Study participants of the Latino communities also identified traumatic experiences as factors that influenced health. In contrast to the study participants of the American Indian community, who emphasized family- and community-level sources of trauma, the study participants of the Latino communities emphasized personal-, family-, and community-level sources of trauma. At the personal level, trauma was experienced during the immigration experience. Carolina, a woman with over ten years’ experience working for a health center in the Latino community shared,

But many of them [immigrants], you know, come from Mexico. Many of them, they get arrested. Some people experience bad [things] when they are crossing the [border] or they experience being sexual[ly] assault[ed]. Other experiences, who knows. There are so many things they’ve been collecting on their way here, to this dream, you know, is going to affect their lifestyle and is going to affect their health.

At the family level, examples of trauma were similar to those identified in the American Indian community, such as personal violence. One participant discussed the manner in which present-day trauma affected the health of her community. She stated,

Oftentimes, as the kids see and witness domestic violence in the home, they later in life kinda turn things against mom…so there’s a lot of factors that come into play when it comes to literally living a healthy lifestyle.

Examples of trauma at the community level included gang violence and drug dealing. While study participants of the American Indian community also identified these problems in the community, community-level trauma was most often discussed in terms of historical events and the effect of those historical events on the community over time. Thus, the Latino and American Indian communities differed in the ways that trauma was experienced by residents; nonetheless, trauma as a source of poor health or a barrier to good health emerged as a cross-cutting theme among all study participants.

Some study participants in all communities believed that discrimination in the community was a form of trauma that persisted in their communities, which some or all community residents endured. While participants’ discussions of discrimination were set in different contexts for each community, the discussions consistently pointed to the negative health effects of discrimination.

#### Invisibility

The third theme that emerged from our analyses of the interview transcripts was that some residents were invisible in the community, making them especially vulnerable to poor health. Study participants believed that some residents were not valued or respected in the work place, did not have representation in government or policy agenda-setting activities, or were not treated with respect and/or dignity in their communities in general, or in specific settings such as medical offices. The following interview excerpts illustrated this notion of invisibility:

…Often workers talk about feeling as though [in the] places that they work, the livestock or the crops are more valued than they are. I mean not everyone speaks in this sense, but it’s a common sense in many workers. – Participant from the rural Latino community

…A lot of it is the elders in our community don’t have transportation or because of their health situation…they need to be seen by a doctor…and not be overlooked…That’s the kind of concern that I’m getting calls [about]. – Participant from the American Indian community

Although these excerpts referred to unique situations of each community, they both reflected a similar notion of invisibility. These discussions by study participants also suggested that being invisible in the community created a sense of powerlessness among residents and damaged a person’s health. When asked if the migrant seasonal farmworkers in the rural Latino community were healthy, one participant answered:

No, because of access, cost, the economics of health care and the fact that they [farmworkers] are really not recognized or appreciated for what they do. They’re looked upon more as just a labor force instead of people…. I see a lot of the men out here, the day laborers that we have, who can’t afford to have good health care. Many of them are undocumented so there’s a lot of places that receive federal funds that can’t assist them because of their documented status.

This excerpt illustrated farmworkers’ invisibility in the workplace and migrant camps and the connection of invisibility to health. In this statement, the participant tied lack of access to unhealthy living conditions and being undocumented, suggesting that these factors, which reflected not having a place in the community, kept the residents from having good health. Similar views were shared by study participants of the other communities.

## Discussion

This study was guided by an ecological framework [8] to examine the multiple levels of factors that influenced the health of residents in three Oregon communities, as perceived by community leaders in those communities. Our content and thematic analyses showed that a range of factors were important to the communities’ health. On an individual level, our content analyses indicated that unity and inclusivity were important to good health. These findings are supported by other research showing the value of social support in pursuing healthy lifestyles [16-20]. At the community level, our content analyses indicated that available health services, safety, physical activity and recreational resources, and accessibility of affordable healthy foods were instrumental to a community’s health. Our results showed that most of the study participants did not consider their communities to be healthy precisely because their communities were deficient in these areas, such as insufficient resources and safety. These findings are consistent with the results from the 2011 Oregon State of Equity Report, which showed disparities between American Indians and non-Latino whites in enhanced child care and preventive services for children covered by the Oregon Health Plan, and disparities in safety net clinic use between Latinos and non-Latino whites [7]. Our study identified other attributes that contributed to poor health, such as poverty, discrimination, and high rates of preventable chronic health conditions among its residents, which have been identified in other vulnerable populations. In particular, other research has identified threats to safety [21-23], poor access to parks and/or exercise facilities [24], and poor access to healthy foods [20,21,23] as correlates of poor community health. Our findings are also supported by recent research in Oregon which found MSFWs experience significant physical and economic barriers to culturally appropriate fruits and vegetables [25].

Our thematic findings also suggest that these different factors are intertwined. For example, our results suggest that trauma and invisibility are important components of American Indian and immigrant Latinos’ perceptions of the causes of poor personal and community health. While the context of trauma and invisibility differed between the participants of the Latino and American Indian communities, the result was the same in that they were perceived as having negative consequences for the community residents personally and for the community as a whole.

The concept of historical trauma was introduced under the purview of psychotherapy in the 1990s with American Indian populations [26]. Historical trauma has been characterized as a collective experience shared by members of an identifiable group, which has psychological and social sequelae of historical oppression and results in negative impacts that accumulate over time [27]. Examples of historical trauma include the forced use of residential schools to drive out the culture among American Indian children [28], slavery among African Americans [29,30], and more recently, immigration among Latino populations [31]. Proponents of historical trauma argue that long-term mass trauma is associated with higher prevalence of physiological and psychiatric disease even several generations after the original trauma occurred [31,32]. While historical trauma theories are relatively new frameworks for public health research [31] and are not without criticism [33], there is a growing body of evidence showing the associations between exposure to historical trauma or current day microaggressions, such as racism, discrimination and daily hassles [34,35], and elevated levels of post-traumatic stress symptoms [36,37], psychiatric problems [38,39], cardiovascular disease [40,41], poor self-rated health [39], alcohol dependency [42], substance abuse [28,38,43], and smoking [44], among other poor health outcomes [45].

Some of the research suggests that response to trauma is transmitted from one generation to another through a genetic adaptation resulting in greater susceptibility to health problems [46] or through poor social behaviors, such as alcohol and drug use [47]. However, these findings are not without controversy. As Green and Darity [33] point out, historical trauma theories as a mechanism for poor health among disenfranchised populations have a compelling limitation in that it is nearly impossible to measure a pure ‘trauma’ effect; there is no definitive time period in which historical trauma ends and current experiences of discrimination and economic hardship begin, and both historical and current trauma are associated with poor health outcomes. Nevertheless, we found that some of the American Indian study participants believed that the negative effects of forced residential schooling in older generations continue to plague their community today. Similarly, we found that Latino study participants believed that trauma from immigration experiences and domestic violence has an ongoing negative effect on the community’s health.

We found that some community residents were perceived as invisible because they were not recognized for their contributions in the work place or because they were not seen by the community at large as having a social role. These findings are consistent with a recent study that documented the unseen labor of migrant farmworkers in southeastern Georgia and how invisibility in multiple public institutions, including health care and social services, contributed to the illness among farmworkers [48]. Our findings are also consistent with the under appreciation of domestic workers [49], grave diggers [50] and commercial custodians [51]. Overall, however, the effects of invisibility or social recognition on health outcomes have received limited attention in the public health literature where invisibility has been studied in terms of disease diagnosis and symptom manifestation of chronic pain, rheumatoid arthritis and fibromyalgia, and cancer [52-55]. However, these findings resonate with the broader concept of marginalization, the process by which specific groups of people are relegated to the fringes of society, thereby creating inequities in multiple life domains, such as health, education, and income, among others [56]. More research is needed to examine the relationship of perceived invisibility to marginalization, whether invisibility is a byproduct of being marginalized or part of the marginalization process itself.

While our findings on invisibility suggest that isolation and exclusion from community life may be damaging to one’s health, our findings on social connectedness may have just the opposite effect. Our findings on the importance of being socially connected suggest that having a place within the family and in the community in general may have consequential health benefits at the individual and community level. A study with a Lakota tribe found that collective identity and commitment to traditionally oriented values and healing can transcend trauma [57], suggesting that group unity may be an important yet overlooked factor in community health. Social connections are also of paramount importance in Latino families and communities. For example, the structure of the traditional Latino family is based on a strong extended-family system which includes fictive kin [58,59] in addition to blood relatives. Studies have found that familism, a dedication to the family as a principle, is an enduring cultural belief in Latino populations [60,61]. Consistent with this literature, we found that family and community unity are viewed as critically important for having a healthy community. Moreover, the influence of social connectedness on health, as perceived by the participants in our study, is underscored in a recent survey of Mexican-origin farmworkers in rural northwest Oregon. Lopez-Cevallos and colleagues found that local providers and institutions such as churches that were considered “trusted” among farmworkers weakened fears about deportation, thereby facilitating access to needed health care and creating a safe environment for undocumented residents [62].

Our study has some limitations. Participants were a self-selected group of highly engaged members of their respective communities. Thus, their views may not represent those of all community residents, although the use of snowball sampling helped us to identify participants who could speak to the many different facets of their communities. The small sample size does not allow for sub-group analyses. However, we sought in this paper to identify universal themes from the entire data, allowing us to focus on similarities rather than differences and to propose findings that warrant further examination. Lastly, most interviews were conducted in person; however several were conducted by telephone, which may have affected the data collected in those interviews.

## Conclusions

Two of Healthy People’s 2020 overarching goals are to “create social and physical environments that promote good health for all” and “promote quality of life, healthy development, and healthy behaviors across all life stages” [63]. Research studies have found that where people live affects their health [5,6]. Our study’s findings provide a richer, more nuanced insight to the social contexts which operate in the lives of some Latinos and American Indians in Oregon. While participants identified community-level factors as important to health, they focused more on the social connections of individuals to each other and the relationships that residents have with their communities at-large. Our findings may also help to explain how the intra- and inter-personal levels, the community/institutional level, and the macro level/public policy contexts can serve to influence the health of Latinos and American Indians. For example, trauma and invisibility are not routinely examined in community health assessments and improvement planning; nonetheless, these factors appear to be at play affecting the health of residents in the three communities in our study.

## References

[1] Macartney S, Bishaw A, Fontenot K (2013). Poverty rates for selected detailed race and Hispanic groups by state and place: 2007–2011. Am Comm Survey Briefs.

[2] Smedley BD, Stith AY, Nelson AR (2009). Unequal treatment: Confronting racial and ethnic disparities in health care (with CD).

[3] Denny CH, Holtzman D, Cobb N (2003). Surveillance for health behaviors of American Indians and Alaska natives. Findings from the behavioral risk factor surveillance system, 1997–2000. Morb Mortality Weekly Report Surv Summ (Washington DC 2002).

[4] Saffer H, Dave DM, Grossman M (2011). Racial, ethnic and gender differences in physical activity.

[5] Bernard P, Charafeddine R, Frohlich KL, Daniel M, Kestens Y, Potvin L (2007). Health inequalities and place: a theoretical conception of neighbourhood. Soc Sci Med.

[6] Cummins S, Curtis S, Diez-Roux AV, Macintyre S (2007). Understanding and representing ‘place’in health research: a relational approach. Soc Sci Med.

[7] Oregon Health Authority (2011). Oregon Department of Human Services:State of Equity Report.

[8] Fitzgerald N, Spaccarotella K (2009). Barriers to a healthy lifestyle: from individuals to public policy—an ecological perspective. J Ext.

[9] County Health Rankings & Roadmaps. Accessed February 4, 2015 [http://www.countyhealthrankings.org/app/oregon/2014/overview]

[10] Cully Neighborhood Fact Sheet [http://www.cullyneighbors.org/InnovEditor/assets/5-2013%20cully_fact_sheet_english.black.pdf]

[11] U.S. Census Bureau. State and County QuickFacts. Accessed December 13, 2014. [http://quickfacts.census.gov/qfd/states/41000.html]

[12] Larsen AC (2013). Oregon Update: Migrant and Season Farmworker Enumeration Profiles Study.

[13] Bernard HR (2006). Research Methods in Anthropology: Qualitative and Quantitative Approaches.

[14] Friese S (2012). Qualitative data analysis with ATLAS. ti.

[15] Strauss AL, Corbin J, Denzin NK, Lincoln YS (1994). Grounded Theory Methodology: An Overview. Handbook of Qualitative Research.

[16] Watt RG, Heilmann A, Sabbah W, Newton T, Chandola T, Aida J (2014). Social relationships and health related behaviors among older US adults. BMC Public Health.

[17] Fukuoka Y, Kamitani E, Bonnet K, Lindgren T (2011). Real-time social support through a mobile virtual community to improve healthy behavior in overweight and sedentary adults: a focus group analysis. J Med Internet Res.

[18] Kaiser BL, Brown RL, Baumann LC (2010). Perceived influences on physical activity and diet in low-income adults from two rural counties. Nurs Res.

[19] Osuji T, Lovegreen SL, Elliott M, Brownson RC (2006). Barriers to physical activity among women in the rural midwest. Women Health.

[20] Russell HA, Rufus C, Fogarty CT, Fiscella K, Carroll J (2013). ‘You need a support. When you don’t have that… chocolate looks real good’. Barriers to and facilitators of behavioural changes among participants of a healthy living program. Fam Pract.

[21] Adams AK, Harvey H, Brown D (2008). Constructs of health and environment inform child obesity prevention in American Indian communities. Obesity.

[22] Chatterjee N, Blakely DE, Barton C (2005). Perspectives on obesity and barriers to control from workers at a community center serving low-income Hispanic children and families. J Community Health Nurs.

[23] Kim LP, Harrison GG, Kagawa-Singer M (2007). Perceptions of diet and physical activity among California Hmong adults and youths. Prev Chronic Dis.

[24] Leiferman J, Swibas T, Koiness K, Marshall JA, Dunn AL (2011). My baby, my move: examination of perceived barriers and motivating factors related to antenatal physical activity. J Midwifery Womens Health.

[25] Grauel K, Chambers KJ (2014). Food deserts and migrant farmworkers: assessing food access in Oregon’s Willamette valley. J Ethnobiol.

[26] Braveheart MY, DeBruyn LM (1998). The American Indian holocaust: healing historical unresolved grief. Am Indian Alsk Native Ment Health Res.

[27] Gone JP (2013). Redressing first nations historical trauma: theorizing mechanisms for indigenous culture as mental health treatment. Transcult Psychiatry.

[28] Braveheart MY (2003). The historical trauma response among natives and its relationship with substance abuse: a Lakota illustration. J Psychoactive Drugs.

[29] Curtin PD (1992). The slavery hypothesis for hypertension among African Americans: the historical evidence. Am J Public Health.

[30] Kaufman JS, Hall SA (2003). The slavery hypertension hypothesis: dissemination and appeal of a modern race theory. Epidemiology.

[31] Sotero MM (2006). A conceptual model of historical trauma: implications for public health practice and research. J Health Disabil Res Pract.

[32] Foster RP (2001). When immigration is trauma: guidelines for the individual and family clinician. Am J Orthopsychiatry.

[33] Green TL, Darity WA (2010). Under the skin: using theories from biology and the social sciences to explore the mechanisms behind the black-white health gap. Am J Public Health.

[34] Evans-Campbell T (2008). Historical trauma in American Indian/native Alaska communities: a multilevel framework for exploring impacts on individuals, families, and communities. J Interpers Violence.

[35] Wing Sue D, Capodilupo CM, Torino GC, Bucceri JM, Holder AMB, Nadal KL (2007). Racial microaggressions in everyday life: implications for clinical practice. Am Psychol.

[36] Kessler RC, Sonnega A, Bromet E, Hughes M, Nelson CB (1995). Posttraumatic stress disorder in the National Comorbidity Survey. Arch Gen Psychiatry.

[37] Yehuda R (2002). Post-traumatic stress disorder. N Engl J Med.

[38] Basáñez T, Unger JB, Soto D, Crano W, Baezconde-Garbanati L (2013). Perceived discrimination as a risk factor for depressive symptoms and substance use among Hispanic adolescents in Los Angeles. Ethnicity Health.

[39] Brondolo E, Hausmann LRM, Jhalani J, Pencille M, Atencio-Bacayon J, Kumar A (2011). Dimensions of perceived racism and self-reported health: examination of racial/ethnic differences and potential mediators. Ann Behav Med.

[40] Cooper RS (2001). Social inequality, ethnicity and cardiovascular disease. Int J Epidemiol.

[41] Sawchuk CN, Roy-Byrne P, Goldberg J, Manson S, Noonan C, Beals J (2005). The relationship between post-traumatic stress disorder, depression and cardiovascular disease in an American Indian tribe. Psychol Med.

[42] Koss MP, Yuan NP, Dightman D, Prince RJ, Polacca M, Sanderson B (2003). Adverse childhood exposures and alcohol dependence among seven Native American tribes. Am J Prev Med.

[43] Ornelas IJ, Hong S (2012). Gender differences in the relationship between discrimination and substance use disorder among Latinos. Subst Use Misuse.

[44] Lorenzo-Blanco EI, Cortina LM (2013). Latino/a depression and smoking: an analysis through the lenses of culture, gender, and ethnicity. Am J Community Psychol.

[45] Williams DR, Neighbors HW, Jackson JS (2003). Racial/ethnic discrimination and health: findings from community studies. Am J Public Health.

[46] Benyshek DC (2005). Type 2 diabetes and fetal origins: the promise of prevention programs focusing on prenatal health in high prevalence native American communities. Hum Organ.

[47] Myhra LL (2011). “It runs in the family”: intergenerational transmission of historical trauma among urban American Indians and Alaska natives in culturally specific sobriety maintenance programs. Am Indian Alsk Native Ment Health Res.

[48] Bail KM, Foster J, Dalmida SG, Kelly U, Howett M, Ferranti EP (2012). The impact of invisibility on the health of migrant farmworkers in the southeastern United States: a case study from Georgia. Nurs Res Pract.

[49] Hochschild AR (2000). The nanny chain. Am Prospect.

[50] Pinheiro F, Fischer FM, Cobianchi CJ (2012). Work of gravediggers and health. Work J Prev Assessment Rehabil.

[51] Hviid K, Smith LH, Frydendall KB, Flyvholm M (2013). Visibility and social recognition as psychosocial work environment factors among cleaners in a multi-ethnic workplace intervention. Int J Environ Res Public Health.

[52] Dow CM, Roche PA, Ziebland S (2012). Talk of frustration in the narratives of people with chronic pain. Chronic Illness.

[53] Evans J, Ziebland S, Pettitt AR (2012). Incurable, invisible and inconclusive: watchful waiting for chronic lymphocytic leukaemia and implications for doctor-patient communication. Eur J Cancer Care.

[54] Jefferies H, Clifford C (2012). Invisibility: the lived experience of women with cancer of the vulva. Cancer Nurs.

[55] Kool MB, van Middendorp H, Bijlsma JW, Geenen R (2011). Patient and spouse appraisals of health status in rheumatoid arthritis and fibromyalgia: Discrepancies and associations with invalidation. Clin Exp Rheumatol.

[56] Ruano AL, Sanchez S, Jerez FJ, Flores W (2014). Making the post-MDG global health goals relevant for highly inequitable societies: findings from a consultation with marginalized populations in Guatemala. Int J Equity Health.

[57] Braveheart MY (2000). Wakiksuyapi: carrying the historical trauma of the Lakota. Tulane Stud Soc Welfare.

[58] Keefe SE (1984). Real and ideal extended familism among Mexican Americans and Anglo Americans: on the meaning of“ close” family ties. Hum Organ.

[59] Sabogal F, Marín G, Otero-Sabogal R, Marín BV, Perez-Stable EJ (1987). Hispanic familism and acculturation: what changes and what doesn’t?. Hisp J Behav Sci.

[60] Gelman CR (2014). Familismo and its impact on the family caregiving of Latinos with Alzheimer’s disease: a complex narrative. Res Aging.

[61] Shurgot GR, Knight BG (2005). Influence of neuroticism, ethnicity, familism and social support on perceived burden in dementia caregivers: pilot testof the transactional stress and social support model. J Gerontol B Psychol Sci.

[62] López-Cevallos DF, Lee J, Donlan W (2014). Fear of deportation is not associated with medical or dental careuse among Mexican-origin farmworkers served by a federally-qualified health center–faith-based partnership: an exploratory study. J Immigr Minor Health.

[63] U.S. Department of Health and Human Services (2010). Healthy People 2020 Framework.

